# The role of mTOR signaling in the regulation of protein synthesis and muscle mass during immobilization in mice

**DOI:** 10.1242/dmm.019414

**Published:** 2015-09-01

**Authors:** Jae-Sung You, Garrett B. Anderson, Matthew S. Dooley, Troy A. Hornberger

**Affiliations:** 1 Program in Cellular and Molecular Biology, University of Wisconsin - Madison, 2015 Linden Drive, Madison, WI 53706, USA; 2 Department of Comparative Biosciences, School of Veterinary Medicine, University of Wisconsin - Madison, 2015 Linden Drive, Madison, WI 53706, USA

**Keywords:** Skeletal muscle, Disuse, Atrophy, Rapamycin, Contraction, Rheb

## Abstract

The maintenance of skeletal muscle mass contributes substantially to health and to issues associated with the quality of life. It has been well recognized that skeletal muscle mass is regulated by mechanically induced changes in protein synthesis, and that signaling by mTOR is necessary for an increase in protein synthesis and the hypertrophy that occurs in response to increased mechanical loading. However, the role of mTOR signaling in the regulation of protein synthesis and muscle mass during decreased mechanical loading remains largely undefined. In order to define the role of mTOR signaling, we employed a mouse model of hindlimb immobilization along with pharmacological, mechanical and genetic means to modulate mTOR signaling. The results first showed that immobilization induced a decrease in the global rates of protein synthesis and muscle mass. Interestingly, immobilization also induced an increase in mTOR signaling, eIF4F complex formation and cap-dependent translation. Blocking mTOR signaling during immobilization with rapamycin not only impaired the increase in eIF4F complex formation, but also augmented the decreases in global protein synthesis and muscle mass. On the other hand, stimulating immobilized muscles with isometric contractions enhanced mTOR signaling and rescued the immobilization-induced decrease in global protein synthesis through a rapamycin-sensitive mechanism that was independent of ribosome biogenesis. Unexpectedly, the effects of isometric contractions were also independent of eIF4F complex formation. Similar to isometric contractions, overexpression of Rheb in immobilized muscles enhanced mTOR signaling, cap-dependent translation and global protein synthesis, and prevented the reduction in fiber size. Therefore, we conclude that the activation of mTOR signaling is both necessary and sufficient to alleviate the decreases in protein synthesis and muscle mass that occur during immobilization. Furthermore, these results indicate that the activation of mTOR signaling is a viable target for therapies that are aimed at preventing muscle atrophy during periods of mechanical unloading.

## Abstract (precis)

**Summary:** The activation of mTOR signaling is both necessary and sufficient to alleviate the decreases in protein synthesis and muscle mass that occur during immobilization.

## INTRODUCTION

Skeletal muscle comprises approximately 40% of total body mass, and the loss of its mass is highly associated with a low quality of life, an increased risk of morbidity and mortality, as well as elevated healthcare costs (Janssen et al., 2004; Srikanthan and Karlamangla, 2011, 2014; Zhou et al., 2010). Importantly, a profound loss of skeletal muscle mass can occur during various conditions that result in mechanical unloading (i.e. disuse atrophy). For example, most orthopedic injuries and non-orthopedic diseases require immobilization of body parts and bed rest, respectively, and, during the initial 2-3 weeks of this period, a rapid disuse atrophy occurs at a rate of approximately 0.5% of total muscle mass per day (de Boer et al., 2007; Wall and van Loon, 2013). Thus, the development of therapies that are aimed at preserving muscle mass during mechanical unloading is of great clinical and fiscal significance.

Skeletal muscle mass is ultimately determined by the balance between the rate of protein synthesis and protein degradation (Goodman et al., 2011c). For instance, a net decrease in protein synthesis and/or a net increase in protein degradation can lead to disuse atrophy. Indeed, both decreased rates of protein synthesis and increased rates of protein degradation have been observed in several animal models of disuse atrophy (Bodine, 2013). Similarly, decreased rates of protein synthesis have also been observed in numerous human models of disuse atrophy, but whether changes in the rate of protein degradation contribute to the atrophic response in humans is less clear (Rennie et al., 2010; Wall and van Loon, 2013). As such, it has generally been concluded that disuse atrophy is primarily driven by a decrease in the rate of protein synthesis, and thus, preventing the decline in protein synthesis could be a viable target for therapies that are aimed at preventing disuse atrophy (Rennie et al., 2010; Wall and van Loon, 2013).

Previous studies have shown that a variety of stimuli, such as nutrients, growth factors and mechanical loading, can regulate protein synthesis in skeletal muscle and this regulation occurs primarily at the level of translation initiation (Kimball and Jefferson, 2010). Furthermore, the regulation of translation initiation by these stimuli is largely mediated by a protein kinase called the mammalian (or the mechanistic) target of rapamycin (mTOR), which exists in at least two characteristically distinct complexes: (a) the rapamycin-sensitive mTOR complex 1 (mTORC1), and (b) the rapamycin-insensitive mTOR complex 2 (mTORC2) (Ma and Blenis, 2009). Numerous studies have shown that signaling by mTORC1, and/or an unidentified rapamycin-sensitive form of mTOR (collectively referred to as mTOR hereafter unless otherwise noted), regulates cap-dependent initiation of translation through the phosphorylation of substrates such as eukaryotic initiation factor (eIF) 4E binding protein 1 (4E-BP1) and p70 ribosomal protein S6 kinase (p70^S6k^). For example, the phosphorylation of 4E-BP1 can promote translation initiation by enhancing the formation of the elF4F complex, which, in turn, recruits the 43S preinitiation complex to the 5′ cap of most mRNAs (Haghighat et al., 1995; Hara et al., 1997). Moreover, phosphorylated and activated p70^S6k^ can promote an increase in the helicase activity of eIF4A, a component of eIF4F, and thus provide an additional stimulus for translation initiation (Raught et al., 2004). Finally, it has been demonstrated that the activation of mTOR via the overexpression of Rheb, a direct activator of mTOR, is sufficient to induce an increase in p70^S6k^ phosphorylation, cap-dependent translation and protein synthesis in skeletal muscles with normal activity (Goodman et al., 2011b, 2010). Therefore, the control of translation initiation by mTOR is considered to be one of the key steps for the regulation of protein synthesis in skeletal muscle.

**TRANSLATIONAL IMPACT**

**Clinical issue**

Immobilization is a complication that arises during many primary conditions (e.g. casting, bed rest etc.) and it results in mechanical unloading of skeletal muscles. In response to mechanical unloading, skeletal muscles undergo a rapid loss of muscle mass, referred to as disuse atrophy. Severe disuse atrophy prolongs the rehabilitation period and negatively impacts the quality of life of individuals subjected to orthopedic- and non-orthopedic-disease-related immobilization. Hence, various therapeutic interventions that can be used to prevent disuse atrophy are being actively explored. Importantly, the rationale behind most of these therapies is based on our current understanding of molecular mechanisms that mediate the atrophic response. Accordingly, several hypothetical mechanisms have been proposed, and one that has received a large amount of interest predicts that mechanical unloading decreases anabolic mTOR signaling. However, the actual role of mTOR in the regulation of muscle mass during mechanical unloading has remained largely undefined.

**Results**

In this study, the authors investigated the potential role of mTOR in the regulation of protein synthesis and muscle mass during mechanical unloading. To accomplish this, the authors first characterized key features of disuse atrophy by employing a new mouse model of hindlimb immobilization. Unexpectedly, they found that immobilization activates anabolic mTOR signaling while simultaneously decreasing protein synthesis and muscle mass. Then, with the use of an mTOR-specific inhibitor, the authors demonstrate that the immobilization-induced activation of mTOR signaling helps to alleviate the decreases in protein synthesis and muscle mass. Moreover, the authors demonstrate that the positive effects of mTOR signaling can be enhanced when mTOR is further activated by mechanical or molecular means. The authors also provide mechanistic insights that indicate that the positive effects of mTOR are primarily mediated by an increase in protein translation efficiency.

**Implications and future directions**

One of the major conclusions from this study is that immobilization-induced decreases in protein synthesis and muscle mass are primarily mediated by an mTOR-independent mechanism. This conclusion directly challenges the currently favored hypothesis and illustrates that future studies that are aimed at understanding mechanisms of disuse atrophy should be focused on defining the mTOR-independent mechanism. In addition to this fundamental conclusion, the authors have also convincingly demonstrated that, during immobilization, the activation of mTOR signaling can prevent declines in protein synthesis and muscle mass. This observation is particularly important because it illustrates that activation of mTOR could be a viable target for therapies that are aimed at preventing disuse atrophy.

A direct link between mTOR signaling and the regulation of protein synthesis and muscle mass has been well documented in models of elevated mechanical loading. For instance, it has been shown that rapamycin, a highly specific inhibitor of mTOR signaling, can prevent the increases in p70^S6k^ phosphorylation and protein synthesis that are induced by various forms of mechanical loading, such as resistance exercise, blood flow restriction exercise and passive stretch (Drummond et al., 2009; Gundermann et al., 2014; Hornberger et al., 2004; Kubica et al., 2005). It has also been shown that rapamycin can prevent chronic mechanical-overload-induced increases in fiber size (i.e. hypertrophy) (Bodine et al., 2001; Goodman et al., 2011a). Based on these points, it has become widely accepted that rapamycin-sensitive mTOR signaling plays a central role in the regulation of protein synthesis and muscle mass during periods of increased mechanical loading. However, the potential role of mTOR signaling in the regulation of protein synthesis and muscle mass during mechanical unloading has remained largely undefined. Therefore, the primary goal of this study was to define the role of mTOR signaling in the regulation of protein synthesis and muscle mass during mechanical unloading. More specifically, using a new mouse model of hindlimb immobilization, we attempted to: (1) characterize immobilization-induced changes in mTOR signaling, protein synthesis and muscle mass, (2) define the role of mTOR in these changes, and (3) determine whether forced activation of mTOR signaling can rescue the decline in protein synthesis and fiber size that is observed in immobilized muscles. Combined, the results from our study have demonstrated that, during immobilization, the activation of mTOR signaling is both necessary and sufficient to alleviate decreases in protein synthesis and muscle mass. Hence, this study highlights that the activation of mTOR signaling could serve as a viable target for therapies that are aimed at preventing atrophy during periods of mechanical unloading.

## RESULTS

### Immobilization decreases the global rates of protein synthesis and muscle mass, but activates mTOR signaling and cap-dependent translation

**Fig. 1.**
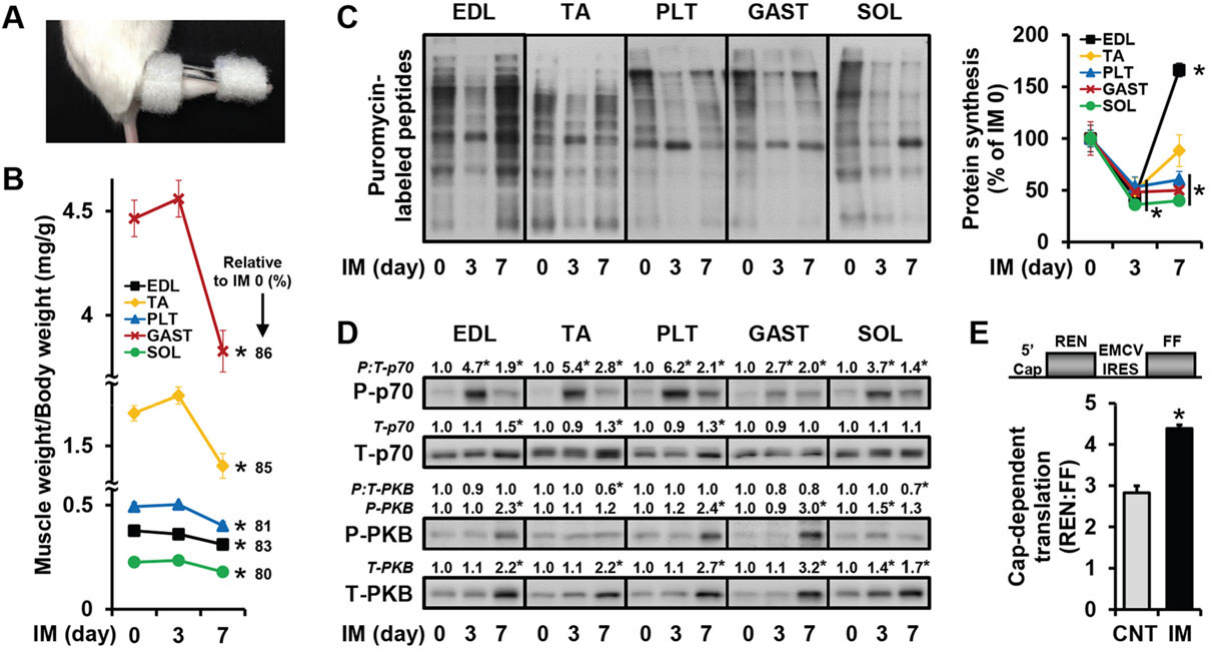
**Immobilization decreases the global rates of protein synthesis and muscle mass, but activates mTOR signaling and cap-dependent translation.** (A) Representative image of the caudal end of a mouse that was subjected to unilateral hindlimb immobilization (IM). (B-D) Mice were subjected to IM for 3 or 7 days, or a non-immobilized control condition (IM 0), and injected with puromycin at 30 min prior to muscle collection for the measurement of protein synthesis by SUnSET. Various lower hindlimb muscles (EDL, extensor digitorum longus; TA, tibialis anterior; PLT, plantaris; GAST, gastrocnemius; SOL, soleus) were weighed to obtain (B) the muscle weight to body weight ratio, and then subjected to western blot analysis for (C) puromycin-labeled peptides, (D) phosphorylated (P) (T389) and total (T) p70, and P (T308)- and T-PKB. The total amount of puromycin-labeled peptides (i.e. protein synthesis), T-p70, P-PKB, T-PKB and P:T ratios of p70 and PKB were expressed relative to the values obtained in the muscle-matched IM 0 control groups. (E) Mouse TA muscles were co-transfected with GFP, and a dual-luciferase bicistronic reporter of cap-dependent translation, and immediately subjected to IM or the non-immobilized control condition (CNT). At 3 days post-transfection, the muscles were collected and luciferase activities produced by cap-dependent translation of *Renilla* luciferase (REN) and cap-independent translation of firefly luciferase (FF) were measured to obtain the REN:FF ratio. All values are presented as the mean (+s.e.m. in graphs, *n*=3-8 muscles per group). * versus the muscle-matched control groups, *P*≤0.05.

In this study, we employed a new mouse model of unilateral hindlimb immobilization that externally immobilizes both the knee and ankle joints in a highly convenient, stable and safe manner (Fig. 1A and supplementary material Movie 1). With this method, we found that the mass of the five major muscles involved in ankle joint movement were significantly reduced after 7 days of immobilization (Fig. 1B) (note: the animal body weight decreases slightly during the first 2 days of immobilization, supplementary material Fig. S1). Furthermore, with the surface sensing of translation (SUnSET) technique, we found that all of the muscles displayed a significant decrease in the amount of puromycin-labeled peptides during the course of immobilization, which demonstrates that immobilization induced a decrease in the global rates of protein synthesis (Fig. 1C). Combined, these results validate the effectiveness of our immobilization model and indicate that the decreases in muscle mass were due, at least in part, to a decrease in the rate of protein synthesis.

Previous studies have shown that the activation of mTOR signaling is necessary for the increases in protein synthesis and muscle mass that occur in response to elevated mechanical loading (Bodine et al., 2001; Drummond et al., 2009; Goodman et al., 2011a; Gundermann et al., 2014; Hornberger et al., 2004; Kubica et al., 2005). Based on these reports, we hypothesized that mechanical unloading with immobilization would lead to a reduction in mTOR signaling and that this, in turn, would contribute to the immobilization-induced decreases in protein synthesis and muscle mass. However, in contrast to this hypothesis, we observed that mTOR signaling, as revealed by the phosphorylated to total ratio of p70^S6k^ (T389), was elevated by immobilization (Fig. 1D). Surprisingly, yet consistent with the role of mTOR in the regulation of cap-dependent translation, we also found that immobilization induced an increase in cap-dependent translation in muscles that had been transfected with a dual-luciferase bicistronic reporter of cap-dependent translation (Fig. 1E) (Goodman et al., 2010). Therefore, these results indicate that immobilization induces a decrease in the global rate of protein synthesis via a mechanism that is independent of mTOR signaling and cap-dependent translation.

Previous studies have demonstrated that an increase in phosphatidylinositol 3-kinase (PI3K)-protein kinase B (PKB) pathway activity can activate mTOR signaling by inhibiting the tuberous sclerosis complex (TSC), which converts active GTP-Rheb into inactive GDP-Rheb (Huang and Manning, 2008). Thus, in an effort to gain insight into the mechanisms through which immobilization activates mTOR signaling, we examined the phosphorylation status of PKB on the T308 residue as a marker of PI3K-PKB pathway activity. As shown in Fig. 1D, the results demonstrated that neither changes in the phosphorylated to total ratio of PKB (an index of PI3K activity) nor the total amount of phosphorylated PKB (a marker of total PKB activity) were correlated with the immobilization-induced activation of mTOR signaling. When combined, these results suggest that the immobilization-induced activation of mTOR signaling is mediated by a PI3K-PKB-independent mechanism.

### Rapamycin exacerbates immobilization-induced decreases in protein synthesis and muscle mass

**Fig. 2.**
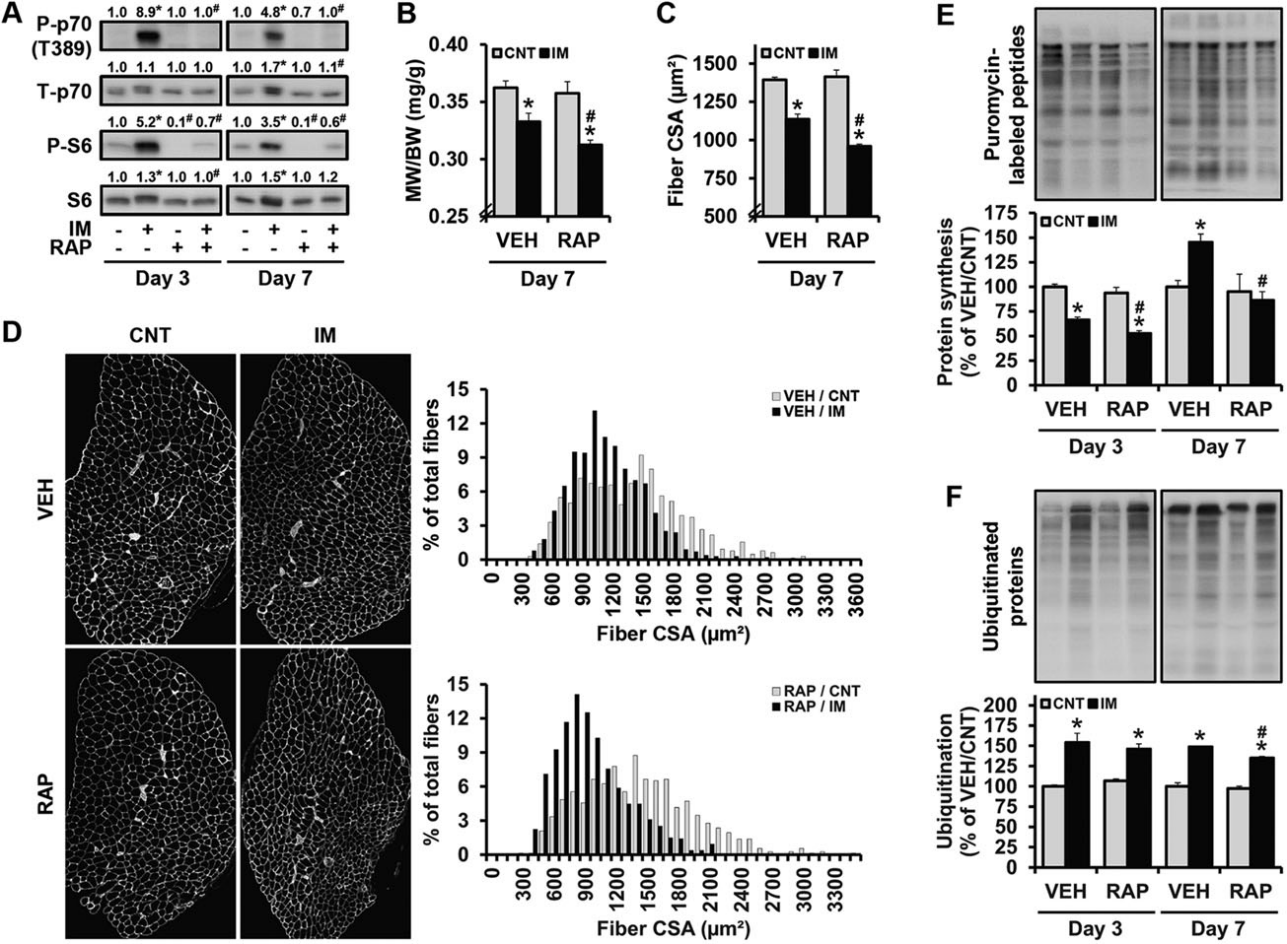
**Rapamycin exacerbates immobilization-induced decreases in protein synthesis and muscle mass.** Mice were subjected to unilateral hindlimb immobilization for 3 or 7 days (IM+), or a non-immobilized control condition (IM− or CNT), and received an acute (day 3) or chronic (day 7) administration of rapamycin (RAP+) or the vehicle (RAP− or VEH) as described in the Materials and Methods. At 30 min prior to the collection of the EDL muscles, mice were injected with puromycin. The muscles were (A) subjected to western blot analysis for phosphorylated (P) (T389) and total (T) p70 and P (S240/244)- and T-S6, (B) analyzed for the muscle weight (MW) to body weight (BW) ratio, (C,D) subjected to immunohistochemistry for laminin to obtain the cross-sectional area (CSA) (≥120 fibers per muscle), or (E,F) subjected to western blot analysis for puromycin-labeled peptides (i.e. protein synthesis) and ubiquitylated proteins, respectively. The values in A, E and F were expressed relative to the values obtained in the time-matched IM−/RAP− (A) or CNT/VEH groups (E,F). All values are presented as the mean (+s.e.m. in graphs, *n*=3-12 muscles per group). * versus the time- and drug-matched IM− or CNT groups, ^#^ versus the time- and mobility-matched RAP− or VEH groups, *P*≤0.05.

Our observation that immobilization induces the activation of mTOR signaling was unexpected, but it was not entirely surprising because recent studies have shown that denervation of the sciatic nerve, which induces neurogenic atrophy, also results in the activation of mTOR signaling (Quy et al., 2013; Tang et al., 2014). However, the functional role of mTOR activation in neurogenic atrophy is not entirely clear because, during denervation, the activation of mTOR signaling not only increases protein synthesis, but it can also increase protein degradation through a negative feedback inhibition of the anti-catabolic PI3K-PKB signaling pathway (Harrington et al., 2005; Quy et al., 2013; Stitt et al., 2004; Tang et al., 2014). Thus, we set out to define the role that mTOR activation plays in immobilization-induced atrophy. To accomplish this, we first performed an experiment in which mice were treated with rapamycin during the period of immobilization. As shown in Fig. 2A, rapamycin effectively inhibited the increase in p70^S6k^ (T389) phosphorylation that was observed in the extensor digitorum longus (EDL) muscles after 3 and 7 days of immobilization. In these analyses, we also examined the phosphorylation status of the ribosomal S6 protein, which is a downstream substrate of p70^S6k^ (Ferrari et al., 1991). As expected, immobilization induced an increase in S6 (S240/244) phosphorylation and rapamycin significantly reduced the phosphorylation of these sites. Moreover, we found that immobilization induced an increase in the total amount of the S6 protein and this event was completely inhibited by rapamycin. Finally, and most importantly, our results demonstrated that rapamycin exacerbates the reductions in muscle mass and fiber size that occur after 7 days of immobilization (Fig. 2B-D).

As mentioned above, the activation of mTOR can increase the rates of protein synthesis and degradation, and, thus, rapamycin could potentially inhibit mTOR-mediated increases in both protein synthesis and degradation during immobilization. Consistent with this suggestion, we found that rapamycin exacerbated the immobilization-induced decrease in the rates of protein synthesis at day 3 (Fig. 2E). Furthermore, our results in Fig. 1C revealed that, after 7 days of immobilization, the rate of protein synthesis in EDL muscles rebounds to a level that is higher than that observed in control muscles and, as shown in Fig. 2E, this effect was attenuated in mice that had been treated with rapamycin. However, in contrast to its clear effects on protein synthesis, rapamycin only slightly inhibited the immobilization-induced increase in global ubiquitylation (a marker for protein degradation), and this effect was only present in muscles that had been subjected to 7 days of immobilization (Fig. 2F). As shown in supplementary material Fig. S2, we also obtained similar results when examining mTOR signaling events, muscle mass, rates of protein synthesis, and global ubiquitylation in gastrocnemius muscles. Therefore, it can be firmly concluded that the immobilization-induced activation of mTOR signaling helps to prevent the loss of muscle mass and this effect is primarily driven through the anabolism-favoring effect of mTOR signaling.

### In immobilized muscles, isometric contractions enhance mTOR signaling and rescue the decrease in protein synthesis via a rapamycin-sensitive mechanism

**Fig. 3.**
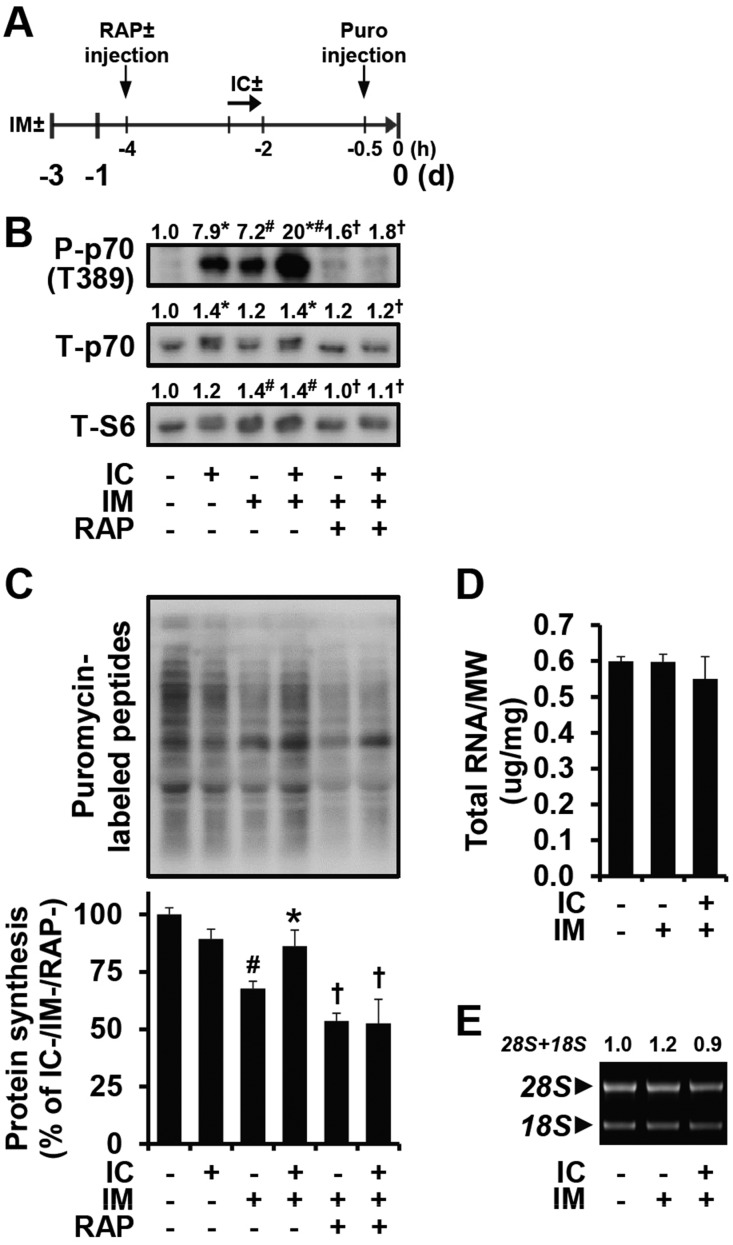
**In immobilized muscles, isometric contractions enhance mTOR signaling and rescue the decrease in protein synthesis via a rapamycin-sensitive mechanism.** (A-C) Mice were subjected to unilateral hindlimb immobilization for 3 days (IM+), or a non-immobilized control condition (IM−), and then injected with rapamycin (RAP+) or the vehicle (RAP−) and puromycin (puro) at the indicated time points. Following the RAP−/+ injections, mice were subjected to a bout of isometric contractions (IC+) or the sham condition (IC−). Upon collection, EDL muscles were subjected to western blot analysis for (B) phosphorylated (P) and total (T) p70, T-S6 and (C) puromycin-labeled peptides (i.e. protein synthesis). (D,E) Mice were treated as in A except for the injections, and the EDL muscles were analyzed for (D) total RNA to muscle weight (MW) ratio and (E) 28S+18S rRNA content. The amount of P-p70, T-p70, puromycin-labeled peptides and 28S+18S rRNA was expressed relative to the values obtained in the IC−/IM−/RAP− groups. All values are presented as the mean (+s.e.m. in graphs, *n*=3-6 muscles per group). * versus the drug- and mobility-matched IC− groups, ^#^ versus the contraction-matched IM−/RAP− groups, ^†^ versus the contraction-matched IM+/RAP− groups, *P*≤0.05.

Previous human studies have shown that electrically evoked contractions can prevent disuse-induced reductions in the rate of protein synthesis and muscle size (Gibson et al., 1988; Hirose et al., 2013). Although the molecular mechanisms behind this effect are not known, current evidence suggests that the activation of mTOR signaling might be involved. For instance, previous studies have shown that the activation of mTOR signaling positively correlates with contraction-induced increases in the rate of protein synthesis and training-induced increases in muscle mass (Baar and Esser, 1999; Kumar et al., 2009; Terzis et al., 2008). Therefore, we reasoned that a further activation of mTOR signaling during immobilization, via electrically evoked contractions, might alleviate the immobilization-induced decrease in protein synthesis. To test this, we treated control and 3-day-immobilized mice with or without an acute bolus of rapamycin and then subjected the mice to a bout of isometric contractions or a sham condition (Fig. 3A). As shown in Fig. 3B, isometric contractions enhanced the level of mTOR signaling in immobilized muscles, and rapamycin abolished this effect. Moreover, in immobilized muscles, isometric contractions enhanced the rate of protein synthesis and, again, this effect was completely abolished by rapamycin (Fig. 3C). Combined, these results indicate that isometric contractions can prevent the immobilization-induced decrease in protein synthesis and this effect is mediated by a rapamycin-sensitive/mTOR-dependent mechanism.

Next, we set out to identify the possible mechanisms that were responsible for the mTOR-dependent effect of isometric contractions on protein synthesis. Although cap-dependent regulation of translation initiation (i.e. translational efficiency) is the best characterized downstream function of mTOR, mTOR can also regulate protein synthesis via changes in translational capacity (Chaillou et al., 2014). Hence, we first measured total RNA levels as a marker of translational capacity. The results of these analyses indicated that total RNA was not affected by isometric contractions (Fig. 3D). Similar results were also obtained when we examined the levels of 28S+18S rRNA and the S6 ribosomal protein (Fig. 3B,E). Taken together, these results suggest that the mTOR-dependent effect of isometric contractions on protein synthesis is mediated through changes in translational efficiency.

### In immobilized muscles, Rheb overexpression enhances mTOR signaling, cap-dependent translation and protein synthesis, and rescues the decrease in fiber size

**Fig. 4.**
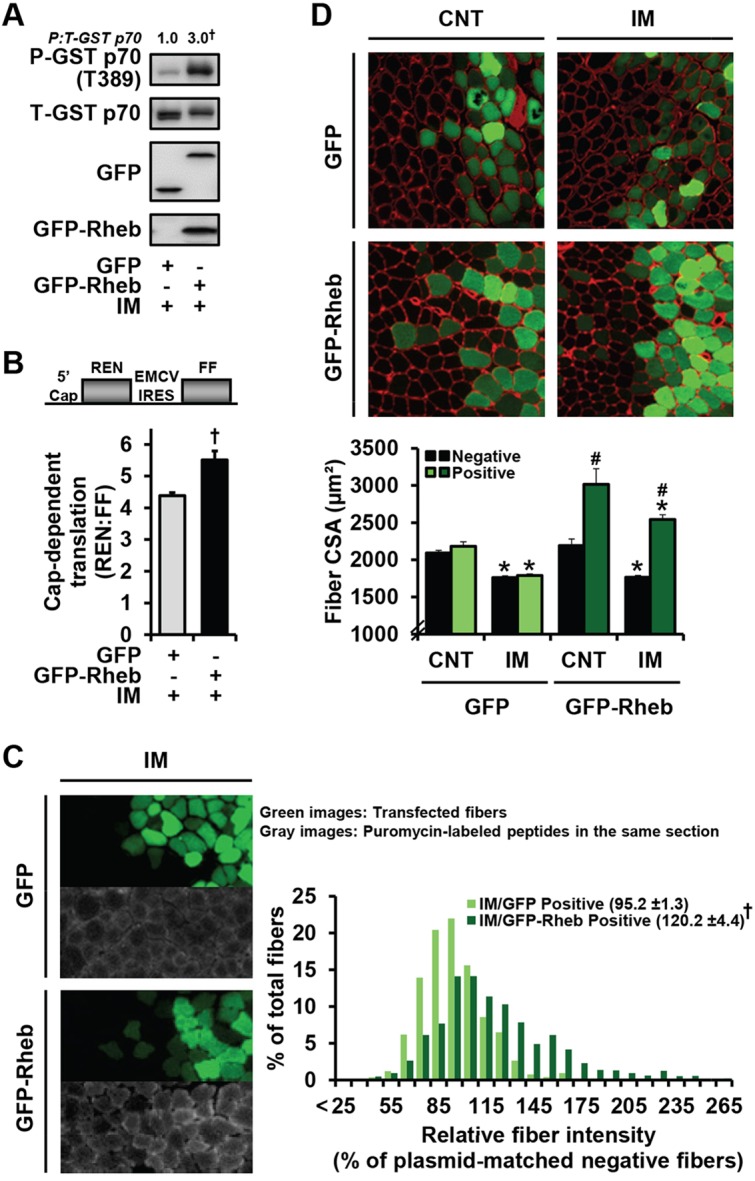
**In immobilized muscles, Rheb overexpression enhances mTOR signaling, cap-dependent translation and protein synthesis, and rescues the decrease in fiber size.** (A) Mouse TA muscles were co-transfected with GST-p70 and GFP or GFP-Rheb, and immediately subjected to unilateral hindlimb immobilization (IM). After 3 days, the muscles were collected and subjected to western blot analysis for phosphorylated (P) and total (T) GST-p70, GFP and GFP-Rheb. The P:T ratio for GST-p70 was expressed relative to the values obtained in GFP (control) group. (B) Mouse TA muscles were co-transfected with a dual-luciferase bicistronic reporter of cap-dependent translation and GFP or GFP-Rheb, and immediately subjected to IM. At 3 days post-transfection, the muscles were collected, and luciferase activities produced by cap-dependent translation of *Renilla* luciferase (REN) and cap-independent translation of firefly luciferase (FF) were measured to obtain the REN:FF ratio. (C) Mouse TA muscles were transfected with GFP or GFP-Rheb, and immediately subjected to IM. After 3 days, the mice were injected with puromycin as in Fig. 1 and the muscles were subjected to immunohistochemistry for GFP and puromycin-labeled peptides. The puromycin staining intensity in transfected (positive) fibers was expressed relative to the value obtained in non-transfected fibers from the same section and plotted on histograms (≥160 fibers per muscle). (D) Mouse TA muscles were transfected with GFP or GFP-Rheb, and immediately subjected to IM or a non-immobilized control condition (CNT). After 7 days, the muscles were collected and subjected to immunohistochemistry for GFP and laminin to obtain the cross-sectional area (CSA) of the transfected (positive) and non-transfected (negative) fibers within each muscle (≥100 fibers per muscle). All values are presented as the mean (+s.e.m. in graphs, *n*=3-4 muscles per group). ^†^ versus GFP groups, * versus the plasmid- and transfection-matched CNT groups, ^#^ versus the mobility-matched GFP-Rheb negative groups as well as the mobility-matched GFP positive groups, *P*≤0.05.

The results from Fig. 3 suggest that, in immobilized muscles, the activation of mTOR signaling can induce an increase in the rate of protein synthesis, which might help to prevent disuse atrophy. To more directly investigate this possibility, we used overexpression of Rheb as a means for inducing the activation of mTOR signaling in immobilized muscles. As shown in Fig. 4A, we confirmed that the overexpression of Rheb in immobilized muscles was sufficient to induce the activation of mTOR signaling, as revealed by an increase in the T389 phosphorylation of co-transfected GST-p70. Furthermore, we determined that the overexpression of Rheb was sufficient to induce an increase in both cap-dependent translation and the global rate of protein synthesis (Fig. 4B,C). Finally, and most significantly, we demonstrated that the overexpression of Rheb robustly increased the size of fibers and prevented the atrophy that occurred during immobilization (Fig. 4D). Combined, these results indicate that the forced activation of mTOR signaling during immobilization can prevent disuse atrophy, at least in part, by promoting an increase in protein synthesis via enhanced cap-dependent translation.

### eIF4F formation is enhanced by immobilization in a rapamycin-sensitive manner, but is not further enhanced by isometric contractions

**Fig. 5.**
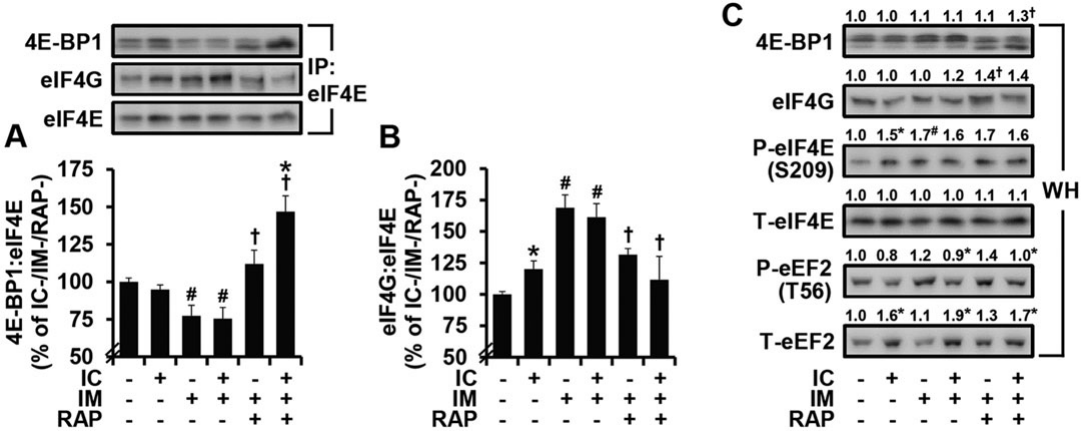
**eIF4F formation is enhanced by immobilization in a rapamycin-sensitive manner, but not further enhanced by isometric contractions.** Mice were treated as in Fig. 3 and pre-cleared homogenates from EDL muscles were subjected to immunoprecipitation (IP) of eIF4E followed by western blot analysis for 4E-BP1, eIF4G and eIF4E to obtain the ratio of (A) 4E-BP1:eIF4E and (B) eIF4G:eIF4E. (C) Whole homogenates (WH) were subjected to western blot analysis for the total (T) and phosphorylated (P) forms of various proteins. All values were expressed relative to the values obtained in the IC−/IM−/RAP− group and presented as the mean (+s.e.m. in graphs, *n*=3-6 muscles per group). * versus the drug- and mobility-matched IC− groups, ^#^ versus the contraction-matched IM−/RAP− groups, ^†^ versus the contraction-matched IM+/RAP− groups, *P*≤0.05. IC, isometric contractions; IM, immobilization; RAP, rapamycin.

To gain insight into the molecular mechanisms through which the activation of mTOR signaling promotes cap-dependent translation/protein synthesis during immobilization, we first examined the effects of immobilization on the association of eIF4E with 4E-BP1 and eIF4G. As described in the Introduction, mTOR-dependent increases in 4E-BP1 phosphorylation can lead to the dissociation of 4E-BP1 from eIF4E while simultaneously enhancing the association of eIF4E with eIF4G (i.e. the formation of eIF4F complex, which is crucial for the cap-dependent initiation of translation). Consistent with these mechanisms, we found that immobilization induced a decrease in the ratio of 4E-BP1:eIF4E and an increase in the ratio of eIF4G:eIF4E, and that these events were sensitive to the inhibitory effects of rapamycin (compare 1st, 3rd and 5th bars from the left in Fig. 5A,B). Interestingly, the immobilization-induced increase in eIF4F formation did not seem to be entirely inhibited by rapamycin, and this effect might be explained by our observation of a rapamycin-insensitive increase in the S209 phosphorylation of eIF4E, which can stabilize the eIF4F complex (Fig. 5C) (Bu et al., 1993). In either case, our results suggest that an mTOR-dependent increase in the formation of the eIF4F complex contributes to the increase in cap-dependent translation and, thereby, an alleviation of the decrease in protein synthesis that occurs during immobilization.

Next, we examined the effects of isometric contractions on the association of eIF4E with 4E-BP1 and eIF4G. Unexpectedly, the results revealed that neither the ratio of 4E-BP1:eIF4E nor the ratio of eIF4G:eIF4E was altered by isometric contractions in immobilized muscles (compare the 3rd and 4th bars from the left in Fig. 5A,B). Therefore, we examined another rapamycin-sensitive regulatory event in protein synthesis, which involves the eukaryotic elongation factor 2 (eEF2). eEF2 is a GTP-binding translation elongation factor that loses its ability to bind to the ribosome (i.e. inactivation) when phosphorylated on the T56 residue by eEF2 kinase (eEF2K) (Carlberg et al., 1990). Importantly, the activity of eEF2K can be negatively regulated by mTOR (Wang et al., 2001). Hence, the activation of mTOR should lead to an increase in the amount of T56-non-phosphorylated eEF2 and, in turn, an increase in the rate of protein synthesis. As shown in Fig. 5C, we found that isometric contractions led to a decrease in the amount of T56-phosphorylated eEF2 along with an increase in the amount of total eEF2. Thus, these results indicate that isometric contractions promote an increase in the amount of T56-non-phosphorylated eEF2; however, this effect was not inhibited by rapamycin (Fig. 5C). When taken together, our results suggest that, in immobilized muscles, the isometric-contraction-induced increase in protein synthesis is mediated through a unique rapamycin-sensitive/mTOR-dependent mechanism that does not involve changes in eIF4F formation or eEF2 activity.

### Immobilization induces fiber-type-dependent decreases in protein synthesis and fiber size that are not associated with the level of mTOR activity

**Fig. 6.**
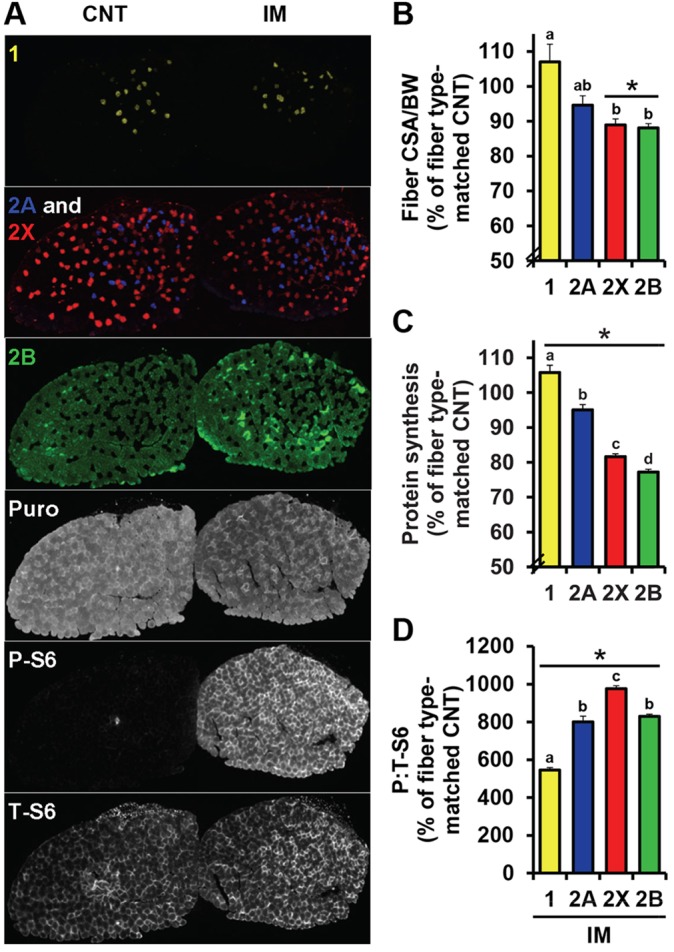
**Immobilization induces fiber-type-dependent decreases in protein synthesis and fiber size that are not associated with the level of mTOR activity.** Mice were subjected to unilateral hindlimb immobilization (IM) for 3 days, or a non-immobilized control condition (CNT), and injected with puromycin as in Fig. 1. (A) EDL muscles obtained from CNT and IM mice were frozen adjacent to one another and subjected to immunohistochemistry for different isoforms of myosin heavy chain (MHC; 1, yellow; 2A, blue; 2X, red; 2B, green) and puromycin-labeled peptides, phosphorylated (P)-S6 (S240/244), or total (T)-S6. (B-D) Fiber-type-specific measurements of (B) the fiber cross-sectional area (CSA) normalized to body weight (BW), (C) the puromycin staining intensity (i.e. protein synthesis), and (D) the ratio of P-S6 normalized to T-S6. All values were expressed relative to the values obtained in the fiber-type-matched CNT muscles and presented as the mean+s.e.m. (*n*=48-360 fibers per group from four independent pairs of muscles). * versus the fiber-type-matched CNT groups, a-d versus one another, *P*≤0.05.

We have previously shown that the regulation of fiber size in response to mechanical overload and food deprivation varies among different fiber types, and that this variation is associated with similar fiber-type-dependent alterations in the rate of protein synthesis and mTOR activity as revealed by S6 (S240/244) phosphorylation (Goodman et al., 2012). Furthermore, in Fig. 2D, immobilization seemed to preferentially reduce the size of larger fibers. Hence, we wondered whether changes in fiber size and protein synthesis during immobilization are also fiber-type-dependent, and whether these changes are associated with similar alterations in the level of mTOR signaling. Specifically, we hypothesized that the magnitude of any fiber-type-dependent decreases in protein synthesis would be inversely correlated with the magnitude of mTOR activation. As shown in Fig. 6B, we first determined that immobilization decreases the size of fibers in EDL muscles with the following fiber-type-dependent order: 1≤2A<2X=2B. Interestingly, it was also found that immobilization decreases the rates of protein synthesis in a similar fiber-type-dependent fashion with type 1<2A<2X<2B (Fig. 6C) [note: similar results were also observed in tibialis anterior (TA) muscles; data not shown]. On the other hand, we were not able to detect an inverse correlation between the fiber-type-dependent regulation of protein synthesis and the level of mTOR signaling as revealed by S6 (S240/244) phosphorylation (Fig. 6D). Therefore, these results suggest that: (1) the regulation of protein synthesis during immobilization largely influences the extent of disuse atrophy, and (2) the fiber-type-dependent decreases in the rate of protein synthesis are not mediated through fiber-type-dependent differences in the regulation of mTOR signaling.

## DISCUSSION

The maintenance of skeletal muscle mass contributes substantially to health and issues associated with the quality of life. However, many primary conditions necessitate the immobilization of body parts, which results in mechanical unloading. During mechanical unloading, skeletal muscles undergo rapid disuse atrophy and this occurs primarily through a decrease in the rate of protein synthesis (Phillips et al., 2009; Rennie et al., 2010). Hence, defining how mechanical unloading impairs protein synthesis and induces atrophy has remained a long-standing question. Accordingly, several hypotheses have been proposed to explain this phenomenon, and one that has received a large amount of interest suggests that a decrease in anabolic mTOR signaling might be involved (Bodine, 2013; Kelleher et al., 2013). Indeed, previous animal studies have shown that various forms of mechanical unloading, such as hindlimb immobilization and hindlimb suspension, can induce decreases in mTOR signaling and protein synthesis (Hornberger et al., 2001; Kelleher et al., 2013; You et al., 2010). Yet, several human studies with immobilization have casted doubt on this hypothesis because they observed decreases in the rates of protein synthesis and muscle mass in the absence of changes in mTOR signaling (de Boer et al., 2007; Marimuthu et al., 2011). Thus, it seems that, at least in humans, an mTOR-independent pathway plays a predominant role in the regulation of protein synthesis and muscle mass during mechanical unloading. In this study, we provide several lines of evidence that support this possibility by identifying that: (1) immobilization decreases the rate of protein synthesis and muscle mass while simultaneously increasing mTOR activity, and (2) the activation of mTOR via Rheb overexpression can robustly increase the size of fibers in immobilized muscles, but Rheb-overexpressing fibers still undergo immobilization-induced atrophy to almost the same extent as GFP-overexpressing and non-transfected fibers. This latter observation is particularly noteworthy because it strongly suggests that the mechanism through which immobilization reduces muscle size is primarily mediated by a pathway that negatively affects the balance between protein synthesis and degradation in a manner that is parallel to the mTOR signaling pathway.

To date, the identity of the mTOR-independent pathway that drives mechanical-unloading-induced declines in protein synthesis and/or muscle mass remains unknown; however, our observation that immobilization induces fiber-type-dependent decreases in the rates of protein synthesis and fiber size might provide some clues. For example, it has been demonstrated that more oxidative fiber types have higher levels of peroxisome proliferator-activated receptor γ coactivator 1α (PGC1α) than glycolytic fiber types, and that overexpression of PGC1α inhibits the expression of atrophy-related genes as well as denervation- and fasting-induced muscle atrophy (Lin et al., 2002; Russell et al., 2003; Sandri et al., 2006). Hence, in our study, it is possible that lower levels of PGC1α expression in type 2X and 2B fibers might have rendered these fiber types more susceptible to the immobilization-induced atrophy than type 1 and 2A fibers. Likewise, it is tempting to speculate that decreases in PGC1α expression during mechanical unloading might drive the mTOR-independent atrophic response. Indeed, it has been found that the expression of PGC1α is dramatically reduced in response to immobilization and denervation (Kang and Ji, 2013; Sandri et al., 2006). Therefore, examining molecules that are associated with fiber-type-specific expression, such as PGC1α, might be worthy of further investigation for studies that are aimed at identifying the mTOR-independent pathway.

As described above, several lines of evidence indicate that reduced mTOR signaling is not necessary for immobilization-induced decreases in protein synthesis and muscle mass. However, previous studies have also suggested that enhanced mTOR signaling can mitigate the negative effects of mechanical unloading. For example, it has been shown that the administration of clenbuterol, a β2 adrenergic agonist that activates PKB-mTOR signaling, can alleviate hindlimb suspension-induced losses in muscle mass and that this effect is mediated through a rapamycin-sensitive mechanism (Kline et al., 2007). Recent studies have also shown that the administration of tomatidine and capsaicin, natural small molecules that stimulate mTOR signaling, can increase muscle mass in immobilized and hindlimb-suspended muscles, respectively (Dyle et al., 2014; Ito et al., 2013). Finally, the knockdown of Deptor expression, an endogenous inhibitor of both mTORC1 and mTORC2, has been shown to increase the rates of protein synthesis and muscle mass in immobilized muscles (Kazi et al., 2011). The results of our study are in agreement with these findings and provide further support for, and additional mechanistic insight into, the positive role that mTOR signaling can play. Specifically, we have provided evidence that indicates that the immobilization-induced activation of mTOR promotes an increase in eIF4F formation and cap-dependent translation, and that these events, in turn, help to alleviate immobilization-induced decreases in protein synthesis and muscle mass. We have also demonstrated that the positive effects of mTOR signaling can be enhanced when mTOR is further activated by isometric contractions or the overexpression of Rheb. In other words, our results clearly illustrate that the activation of mTOR signaling should be considered as a viable target for therapies that are aimed at preserving muscle mass during periods of mechanical unloading.

The above results clearly demonstrate that the activation of mTOR signaling can positively regulate protein synthesis and muscle mass during mechanical unloading; however, as mentioned in the results, the activation of mTOR can also promote protein degradation through multiple mechanisms (Harrington et al., 2005; Stitt et al., 2004; Zhang et al., 2014). Indeed, a recent study showed that the activation of mTOR signaling that occurs during denervation strongly contributed to the increase in catabolic signaling events such as global ubiquitylation, whereas its contribution to the increase in anabolic signaling events was relatively small (Tang et al., 2014). Consistent with the catabolism-favoring effect of mTOR signaling, this study also showed that the denervation-induced activation of mTOR signaling exacerbated the atrophic response (i.e. neurogenic atrophy). Despite this potential role of mTOR activation in promoting protein degradation, our results demonstrated that the immobilization-induced activation of mTOR signaling contributes only marginally, if at all, to the increase in catabolic pathway, as opposed to its strong anabolic effect on protein synthesis. Therefore, we have concluded that the anti-atrophic effect of mTOR activation in immobilized muscles is primarily driven through the anabolism-favoring effect of mTOR signaling. It is not known what factors determine whether the activation of mTOR will favor anabolism or catabolism; however, given the importance of its final outcome, investigating the switch factors will be an interesting subject for future studies.

In summary, the results from this study demonstrate that: (1) the activation of mTOR signaling that occurs during immobilization helps to alleviate the immobilization-induced decreases in protein synthesis and muscle mass, and (2) the positive effects of mTOR signaling can be enhanced when mTOR is further activated by stimuli such as isometric contractions or the overexpression of Rheb. Therefore, we conclude that the activation of mTOR signaling positively regulates protein synthesis and muscle mass during immobilization. Furthermore, our findings highlight that the activation of mTOR signaling should be considered as a viable target for therapies that are aimed at preventing atrophy during periods of mechanical unloading. In the future, it will be important to define how mTOR signaling is activated during immobilization and how immobilization elicits the mTOR-independent, but fiber-type-dependent, decreases in protein synthesis and muscle size. The resulting knowledge from such investigations should further advance our understanding of the mechanisms through which mechanical unloading regulate muscle mass and might facilitate the development of new therapies for preventing disuse atrophy.

## MATERIALS AND METHODS

### Materials

Rapamycin was purchased from LC laboratories (Woburn, MA) and dissolved in DMSO to generate a 5 mg/ml stock solution. Puromycin was purchased from Calbiochem (EMD Millipore, Billerica, MA) and dissolved in diH_2_O to generate a 75 mM stock solution. Rabbit anti-p70^S6k^, anti-PKB, anti-phospho-PKB (T308), anti-phospho-S6 (S240/244), anti-S6, anti-4E-BP1, anti-eIF4G, anti-eIF4E, anti-phospho-eIF4E (S209), anti-eEF2, anti-phospho-eEF2 (T56) and anti-GFP antibodies were purchased from Cell Signaling (Danvers, MA). Rabbit anti-phospho p70^S6k^ (T389) and mouse anti-Ubiquitin antibodies were purchased from Santa Cruz Biotechnologies (Santa Cruz, CA). Rabbit anti-laminin antibody was purchased from Sigma-Aldrich. Mouse anti-Rheb antibody was purchased from Abnova (Taipei, Taiwan). Mouse anti-eIF4E and mouse anti-puromycin antibodies were obtained from Dr Scot Kimball (Pennsylvania State University, PA) and Dr Philippe Pierre (Centre d'Immunologie de Marseille-Luminy, France), respectively. Mouse control IgG1 antibody was purchased from BioLegend (San Diego, CA). Mouse IgG1 anti-type 2A MHC, mouse IgM anti-type 2B MHC and mouse IgM anti-type 2X MHC antibodies were purchased from the Developmental Studies Hybridoma Bank (Ames, IA). HRP-conjugated anti-rabbit IgG and HRP-conjugated anti-mouse IgG antibodies were purchased from Vector Laboratories (Burlingame, CA). HRP-conjugated anti-mouse IgG Fc 2a, anti-mouse IgG Fab, DyLight-594-conjugated anti-mouse IgG Fc 2a, Alexa-Fluor-488-conjugated anti-mouse IgG1 and AMCA-conjugated anti-mouse IgM antibodies, and normal goat serum, were purchased from Jackson ImmunoResearch Laboratories Inc. (West Grove, PA). Alexa-Fluor-350-conjugated anti-rabbit IgG antibody was purchased from Invitrogen (Carlsbad, CA).

### Plasmid constructs

pEGFP-C3 (GFP) was purchased from Clontech (Mountain View, CA). GFP-tagged Rheb (GFP-Rheb) was generated by replacing the YFP-tag from YFP-Rheb (You et al., 2014) with the GFP tag from the pEGFP-C3. pRK5-myc-p70^S6k^-glutathione transferase (GST-p70^S6k^) was provided by Dr Karyn Esser (University of Kentucky, Lexington, KY). The dual-luciferase bicistronic reporter of cap-dependent translation has been previously described (Carter and Sarnow, 2000) and was obtained from Dr Sunnie Thompson (University of Alabama, Birmingham, AL). All plasmid DNA was grown in DH5α *Escherichia coli*, purified with an EndoFree plasmid kit (QIAGEN, Valencia, CA), and re-suspended in sterile PBS.

### Animals

Eight- to ten-week-old female FVB/N mice (Jackson Laboratories, Bar Harbor, MA) were used for all experiments. The mice were fed *ad libitum* in a room maintained at 25°C with a 12-h:12-h light:dark cycle, and anesthetized with an intraperitoneal (IP) injection of ketamine (100 mg/kg body weight) plus xylazine (10 mg/kg body weight) immediately prior to experimental procedures. In the case of isometric contractions, the mice were anesthetized through inhalation of 1-5% isoflurane in O_2_. At the end of the experimental procedures, muscles were collected and then the mice were euthanized by cervical dislocation under anesthesia. All animal experiments followed protocols approved by the Institutional Animal Care and Use Committee of the University of Wisconsin-Madison (# V01324).

### Immobilization

See Fig. 1A and steps 1-3 in supplementary material Movie 1 for details on how immobilization was conducted. To make a splint, a capless 1.5 ml microfuge tube was cut along its short axis (at 1.2 cm from opening) and the upper cylindrical part was attached to one end of a 3.7-cm-long cut metal paperclip (ACC72585, ACCO Brands, Inc., Lincolnshire, IL) by wrapping them together with the adhesive side of 5×1.1 cm Velcro loop (Velcro 90198, Velcro USA, Inc., Manchester, NH) (Step 1). Then, the other end of the cut paperclip was wrapped with the adhesive side of 3×1.5 cm Velcro loop to enclose the open paperclip region and was used to secure the dorsum of the mouse foot, which will directly contact with the fabric side of the Velcro loop (Step 2). Unilateral mouse hindlimb immobilization was then performed by inserting the right hindlimb into the cylindrical part of the splint and then wrapping the hindfoot and the enclosed end of the paperclip together with another 3×1.5 cm Velcro loop tape (Step 3). The immobilized limbs were maintained with the knee in an extended and the ankle in a plantar-flexed position for 3 or 7 days. Hindlimb muscles from mice that were not placed in the splint served as controls.

### Isometric contractions

Unilateral isometric contractions of the dorsiflexor muscles, such as EDL, were performed by stimulating the sciatic nerve of the right hindlimb, which was maintained in a full plantar-flexed position throughout the stimulation period. The surgical procedures and the pattern of stimulation were performed as previously described (O'Neil et al., 2009). In brief, the sciatic nerve of the right hindlimb was exposed with a small incision and stimulated with 100 Hz, 3-7 V pulses through electrodes that connected the sciatic nerve with a SD9E Grass stimulator (Grass Instruments, Quincy, MA). Each resulting contraction was sustained for 3 s and was followed by a 10 s rest period. This pattern of stimulation was repeated for a total of ten sets of six repetitions with a 1 min rest period between each set. Sham-treated contralateral muscles and sham-treated immobilized muscles served as controls for stimulated muscles in non-immobilized and immobilized animals, respectively.

### Skeletal muscle transfection

Mouse TA muscles were transfected by electroporation as previously described (You et al., 2014). In brief, a small incision was made through the skin covering the TA muscle. A total of 30 µg of plasmid DNA solution containing GFP or GFP-Rheb was then injected into proximal and distal ends (6 µl per injection) of the muscle belly with a 27-gauge needle. In some cases (e.g. co-transfections), the DNA solution also contained 2 µg of GST-p70^S6k^ plasmid DNA. After the injections, two stainless steel pin electrodes (1-cm gap) connected to an ECM 830 electroporation unit (BTX/Harvard Apparatus, Holliston, MA) were laid on top of the proximal and distal myotendinous junctions. Then, eight 20 ms square-wave electric pulses were delivered onto the muscle at a frequency of 1 Hz with a field strength of 160 V/cm. Following the electroporation procedure, the incisions were closed with Vetbond surgical glue and the animals were allowed to recover for 3 or 7 days.

### Rapamycin and puromycin injections

Rapamycin solution was prepared by diluting the appropriate volume of the stock solution needed to inject mice with 1.5 mg/kg body weight in 200 µl of PBS, and subsequently administered into the animals via IP injection. Mice that were injected with an equivalent amount of DMSO diluted in 200 µl of PBS served as vehicle controls. For acute administration, these injections were made 4 h prior to muscle collection. For chronic administration, the injections were made immediately after the immobilization procedure and repeated every 24 h for 7 days. For all *in vivo* measurements of protein synthesis with SUnSET (Goodman et al., 2011b), puromycin solution was prepared by diluting the appropriate volume of the stock solution that was needed to inject mice with 0.04 µmol/g body weight in 200 µl of PBS, and subsequently administered into the animals via IP injection at exactly 30 min prior to muscle collection.

### Sample preparation for immunoprecipitation and western blot analysis

Upon collection, plantaris, gastrocnemius, soleus, EDL and TA muscles were immediately frozen in liquid nitrogen and then stored at −80°C. The frozen muscles were then homogenized with a Polytron in ice-cold buffer A [40 mM Tris (pH 7.5), 1 mM EDTA, 5 mM EGTA, 0.5% Triton X-100, 25 mM β-glycerolphosphate, 25 mM NaF, 1 mM Na_3_VO_4_, 10 μg/ml leupeptin and 1 mM PMSF] and a portion of the whole homogenates was used for western blot analysis as described below. The remaining portion of the whole homogenates was pre-cleared by centrifugation at 10,000 ***g*** for 10 min and the supernatants were used for immunoprecipitation as described below. The protein concentration in the whole homogenates and supernatants was determined with a DC protein assay kit (Bio-Rad, Hercules, CA).

### Immunoprecipitation of eIF4E

Equal amounts of protein from each sample were diluted to the same volume with fresh ice-cold buffer A and then incubated with either mouse anti-eIF4E (1:10) or mouse control IgG antibody for 3 h at 4°C. During this incubation, Protein A agarose beads (Santa Cruz Biotechnologies, Santa Cruz, CA) were blocked with 1% BSA in PBS for 1 h at 4°C and then washed three times with PBS. The antibody-containing samples were then incubated with 20 µl of the blocked beads at 4°C for 3 h. Following the incubation, the beads were pelleted by centrifugation at 500 ***g*** for 30 s and washed four times with fresh ice-cold buffer A. After the washes, the beads were boiled in 1× Laemmli buffer for 5 min and pelleted again. The supernatants were subjected to western blot analysis as described below.

### Western blot analysis

Equal amounts of protein from each sample were boiled in 2× Laemmli buffer and subjected to SDS-PAGE, transfer, blocking, and primary and secondary antibody incubations as previously described (You et al., 2012). The resulting membranes were then developed on film or with a Chemi410 camera mounted to a UVP Autochemi system (UVP, Upland, CA) by using regular ECL (Pierce, Rockford, IL) or ECL Prime (GE healthcare, Piscataway, NJ). Once the appropriate image was captured, the membranes were stained with Coomassie Blue to verify equal loading throughout all lanes. Densitometric measurements of each blot were carried out using ImageJ (NIH).

### Analysis of cap-dependent translation

TA muscles were co-transfected with 30 µg of plasmid DNA encoding either GFP or GFP-Rheb and 5 µg of plasmid DNA encoding a dual-luciferase bicistronic reporter of cap-dependent translation. Collected muscles were homogenized with a Polytron in passive lysis buffer (Promega, Madison, WI), and *Renilla* and firefly luciferase activities were measured with a FLUOstar Optima luminometer (BMG Labtech, Durham, NC) by using the Dual-Luciferase Reporter Assay kit (Promega) as described in the manufacturer's instructions. *Renilla* luciferase activity was normalized to the firefly luciferase activity in the same sample.

### Immunohistochemical analysis of cross-sectional area (CSA), protein synthesis and S6 phosphorylation

Upon collection, muscles from control and immobilized animals were submerged individually (for Fig. 5) or adjacent to one another (for Fig. 6) in optimal cutting temperature (OCT) compound (Tissue-Tek; Sakura, Torrance, CA) at resting length, and frozen in liquid-nitrogen-chilled isopentane. Cross-sections (10-μm thick) from the mid-belly of the muscles were obtained with a cryostat and fixed in acetone for 10 min at −30°C. For experiments that included GFP-transfected muscles, sections were fixed in acetone containing 8% methanol and 2% paraformaldehyde for the first 1 min of the total 10 min fixation period. The sections were then warmed to room temperature for 5 min and rehydrated by incubating in PBS for 15 min. The rehydrated sections were then incubated for 1 h in solution A (PBS containing 0.5% BSA and 0.5% Triton X-100) containing either anti-mouse IgG Fab (1:10; for analysis of puromycin-labeled peptides), normal goat serum (1:20; for analyses of phospho- and total-S6), or none of them (for all conditions not stated above). The sections were then washed with PBS and probed with the indicated primary antibodies dissolved in solution A for 1 h at room temperature. After washing with PBS, the sections were incubated with the appropriate fluorophore-conjugated secondary antibodies dissolved in solution A for 1 h at room temperature. After a final washing with PBS, grayscale signals from each secondary antibody were captured with a DS-QiMc camera mounted on an 80i epifluorescence microscope (both from Nikon, Tokyo, Japan), and the resulting monochrome images were merged with NIS-Elements D image analysis software (Nikon). CSA and signal intensities were then measured by tracing the periphery of randomly selected fibers in each muscle section. All analyses were performed by investigators that were blinded to the sample identification.

### Analysis of total and ribosomal RNA

Frozen muscles were homogenized with an RNAse-free pestle in ice-cold TRIzol (Ambion, Life Technologies, Grand Island, NY) and total RNA was isolated with a PureLink RNA Mini Kit (Ambion) according to the manufacturer's instructions. The concentration of isolated RNA was determined with a NanoDrop 2000 spectrometer (Thermo Scientific, Wilmington, DE) and the amount of total RNA was calculated by multiplying the RNA concentration by the total volume of RNA solution. This value was divided by the muscle weight to obtain µg of total RNA per mg muscle. RNA samples from equivalent amounts of muscle mass were also run on 1% agarose gels to check RNA integrity and measure the ribosomal RNA (rRNA) content. Densitometric measurements of the 28S and 18S rRNA were performed with ImageJ.

### Statistical analysis

All values are expressed as the means (+s.e.m. in graphs). Statistical significance was determined by using the Student's *t*-test (2-tailed, unpaired) or ANOVA (one-way or two-way) followed by post hoc analysis. Differences between groups were considered significant when *P*≤0.05. All statistical analyses were performed on SigmaStat software (San Jose, CA).

## Supplementary Material

Supplementary Material
