# Supplementary material for: The role of mTOR signaling in the regulation of protein synthesis and muscle mass during immobilization in mice
Source: Dis Model Mech. 2015 Sep 1;8(9):1059–69. doi: 10.1242/dmm.019414 (PMC4582099; doi:10.1242/dmm.019414)
Supplement: Supplementary Material [file supp_8_9_1059__index.html]

Supplementary Material 

# The role of mTOR signaling in the regulation of protein synthesis and muscle mass during immobilization in mice

## DMM019414 Supplementary Material

- Supplementary Material
