# Supplementary material for: The role of mTOR signaling in the regulation of protein synthesis and muscle mass during immobilization in mice
Source: Dis Model Mech. 2015 Sep 1;8(9):1059–69. doi: 10.1242/dmm.019414 (PMC4582099; doi:10.1242/dmm.019414)
Supplement: Supplementary Material [file supp_019414_DMM019414supp.pdf]

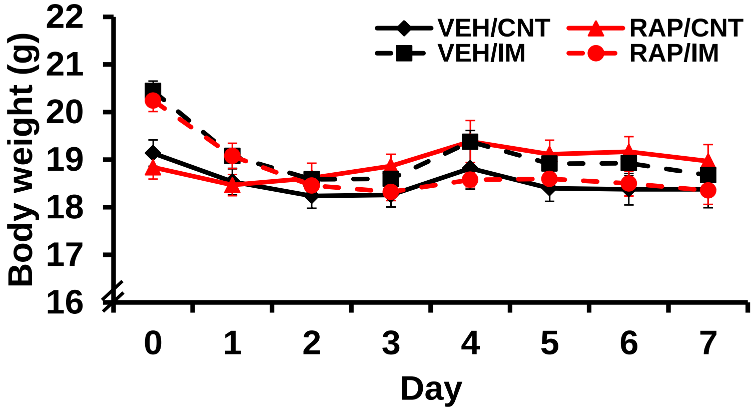

Fig. S1. Time course for changes in body weight during immobilization. Mice were subjected to unilateral hindlimb immobilization for 7 days (IM), or a non-immobilized control condition (CNT), and received a daily administration of rapamycin (RAP, 1.5mg/kg body weight) or the vehicle (VEH). Note: subsequent CSA analyses needed to be performed on samples from animals that had similar final body weights, therefore, the initial animal body weights in the IM groups were intentionally made higher than that in CNT groups.

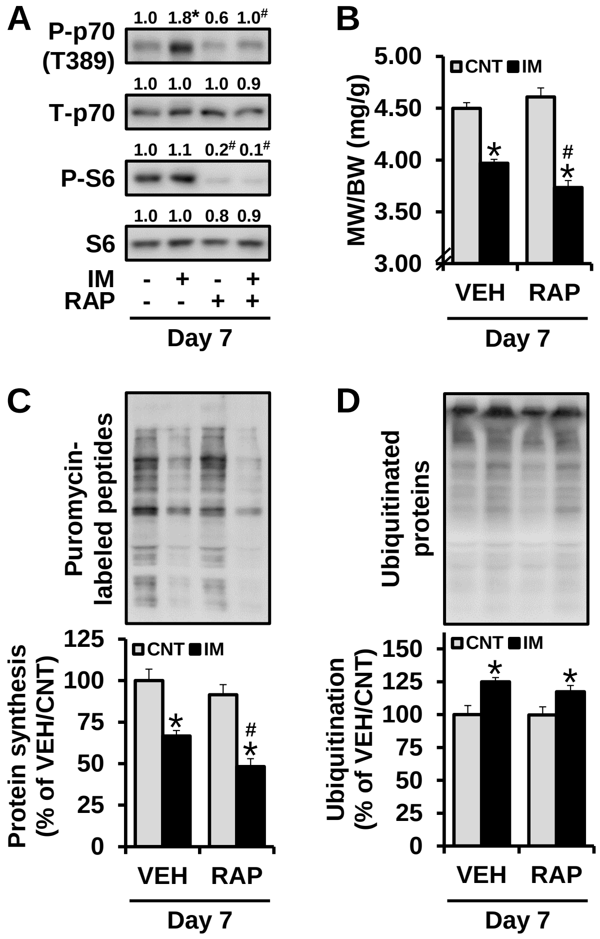

Fig. S2. Rapamycin exacerbates immobilization-induced decreases in protein synthesis and muscle mass. Mice were subjected to unilateral hindlimb immobilization for 7 days (IM+), or a non-immobilized control condition (IM- or CNT), and received a daily administration of rapamycin (RAP+, 1.5mg/kg body weight) or the vehicle (RAP- or VEH). At 30 minutes prior to the collection of the Gastrocnemius muscles, mice were injected with puromycin. The muscles were (**A**) subjected to Western blot analysis for phosphorylated (P) (T389) and total (T) p70 and P (S240/244)- and T-S6, (**B**) analyzed for the muscle weight (MW) to body weight (BW) ratio, or (**C,D**) subjected to Western blot analysis for puromycin-labeled peptides (i.e., protein synthesis) and ubiquitinated proteins, respectively. The values in A, C and D were expressed relative to the values obtained in the time-matched IM-/RAP- (A), or CNT/VEH groups (C,D). All values are presented as the mean (+ s.e.m. in graphs, n = 4-8 muscles per group). * versus the time- and drug-matched IM- or CNT groups, # versus the time- and mobility-matched RAP- or VEH groups, $P \leq 0.05$.

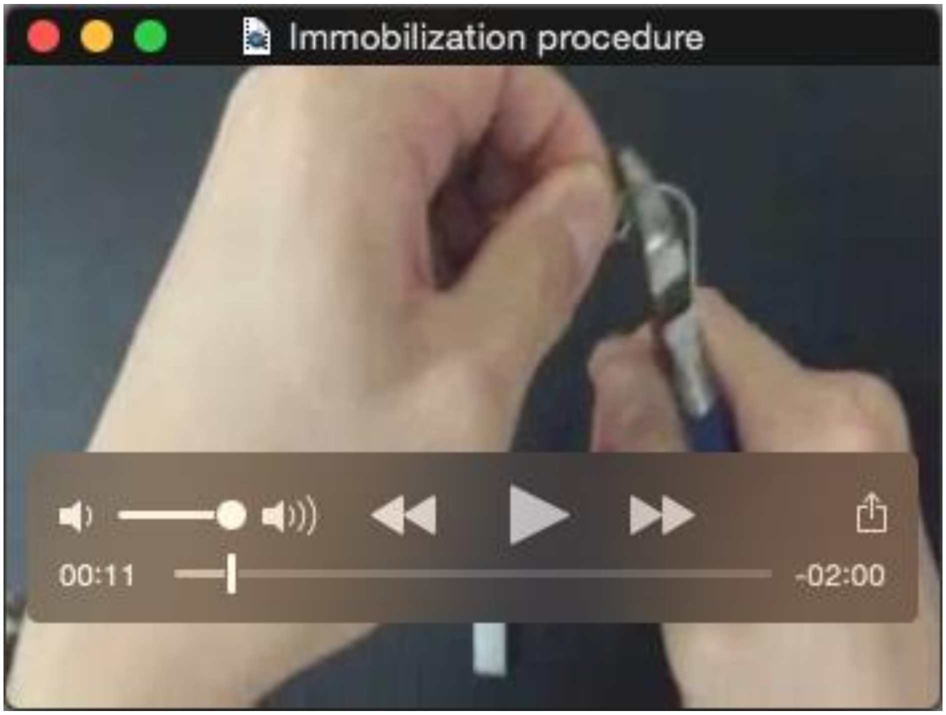

Movie S1. Materials and procedures for a new mouse model of unilateral hindlimb immobilization
